# Primed acclimation of two Greek olive cultivars to water deficit

**DOI:** 10.1007/s12298-026-01747-x

**Published:** 2026-04-30

**Authors:** Georgia-Maria Nteve, Evangelia Stavridou, Emmanouil D. Pratsinakis, Christina Skodra, Michail Michailidis, Stefanos Kostas, Athanassios Molassiotis, Alexios N. Polidoros, Panagiotis Madesis, Irini Nianiou-Obeidat

**Affiliations:** 1 Laboratory of Genetics and Plant Breeding, School of Agriculture, Forestry and Natural Environment, Aristotle University of Thessaloniki, 54124 Thessaloniki, Greece; 2 Department of Botany, School of Biology, Aristotle University of Thessaloniki, 54124 Thessaloniki, Greece; 3 Laboratory of Agronomy, School of Agriculture, Faculty of Agriculture, Forestry and Natural Environment, Aristotle University of Thessaloniki, 54124 Thessaloniki, Greece; 4 Centre for Research and Technology—Hellas, Institute of Applied Biosciences, 6th Km Charilaou-Thermi Rd., 57001 Thessaloniki, Greece; 5 Laboratory of Pomology, Department of Horticulture, Aristotle University of Thessaloniki, 57001 Thessaloniki, Greece; 6 Laboratory of Floriculture, School of Agriculture, Forestry and Natural Environment, Aristotle University, 54124 Thessaloniki, Greece; 7 Laboratory of Molecular Biology of Plants, School of Agricultural Sciences, University of Thessaly, 38446 Volos, Greece

**Keywords:** Drought, Long-term priming, Stress resilience

## Abstract

Water scarcity, intensified by climate change, threatens olive cultivation in the Mediterranean region. Understanding cultivar responses to drought is key to developing strategies to sustain this vital crop. Ιn the present work, we studied the responses of two Greek olive cultivars, "Lefkolia Serron" and "Chondrolia Chalkidikis", to water limitation and we evaluated their ability to acclimatize through cis long term-priming, using a combination of morpho-physiological parameters, primary metabolite profiling and gene expression analysis of drought-related genes. Our findings indicated that the two cultivars follow different strategies to cope with water shortage and priming. Particularly, "Lefkolia Serron" showed a more conservative response, maintaining greater photosynthetic functionality, intrinsic Water Use Efficiency (iWUE) and antioxidant activity upon stress re-exposure. "Chondrolia Chalkidikis" showed greater susceptibility to stress with more pronounced changes at the morpho-physiological, metabolic and gene expression levels. Metabolomic data demonstrated shifts in energy metabolism in response to the drought cycles, while principal component analysis of gene expression patterns showed that drought priming can regulate transcriptional processes, such that drought priming aligned primed plants with controls. Pre-exposure to drought induces a form of transcriptional memory, making the plant more efficient in responding to repeated stress. These findings suggest that long-term drought priming may function as a coping strategy for stressful environmental conditions, however, it is genotype-dependent, as the outcomes vary with genetic background. Understanding olive stress responses and applying eco-friendly strategies like drought priming is a sustainable way to protect this crop from environmental threats.

**Supplementary Information:**

The online version contains supplementary material available at 10.1007/s12298-026-01747-x.

## Introduction

Climate change has led to more frequent and intense environmental conditions, threatening many agricultural systems, including the olive crop (Brito et al. 2019). The Mediterranean region, having the highest proportion of olive cultivation, is increasingly vulnerable to drought and extreme heat (Devot et al. 2023). Notably, over 60% of climate-related agricultural losses in the EU are attributed to drought (Naumann et al. 2021). The Combined Drought Index (CDI) for October 2024 shows that areas in the southern Mediterranean, particularly Italy and Greece, are in a constant state of drought alert, with serious consequences for vegetation (https://joint-research-centre.ec.europa.eu/european-and-global-drought). Despite olives’ moderate drought tolerance, extreme events have already harmed production in these areas (Brito et al. 2019). These conditions result in smaller, dehydrated fruits with lower oil content leading to increased market prices. Beyond water management and adaptive practices, breeding drought-tolerant olive cultivars and understanding their adaptive mechanisms are essential for conserving this emblematic crop in arid regions. Greece, a Mediterranean biodiversity hotspot hosts numerous endemic cultivars yet to be genetically assessed for stress resistance (Bazakos et al. 2023; Tourvas et al. 2023).

Drought tolerance of olive trees is well documented, with several physiological and molecular mechanisms already studied (Fernandes et al. 2018). The main physiological changes in water-deficient plants include leaf curling, stomatal closure, and reduced chlorophyll content (Trabelsi et al. 2019). These modifications, combined with the waxy epidermis of the leaves and dense trichomes, act synergistically to reduce overall water loss (Nteve et al. 2024). Complementary to these above-ground responses, plants also adjust their root-to-shoot ratio, developing deeper and more extensive root systems to maximize water uptake under stressful conditions (Tankari et al. 2021).

Alongside morpho-physiological adjustments, olive trees produce various osmoprotectants, including sugars, proteins, and amino acids (Skodra et al. 2021). Sugars contribute to plant immunity, the so-called "sweet immunity", where monosaccharides such as glucose and fructose, and disaccharides such as sucrose, and trehalose play a critical role in drought tolerance, by maintaining osmotic balance and protecting cellular integrity (Kumar et al. 2021). In addition, amino acids regulate ion channeling, oxidative stress defense, and gene expression pathways (Heidarzadeh 2025). The accumulation of these soluble compounds enhances cellular osmotic pressure, promoting water absorption and thereby maintaining cellular water balance. These physiological and biochemical adjustments are associated with drought-induced gene expression changes that lead to the synthesis of stress-responsive proteins and mRNAs (S. Lisar et al. 2012).

Key genes involved in osmotic adjustment, such as Late Embryogenesis Abundant (*LEA*) and Dehydration-Responsive Element-Binding (*DREB*), regulate the accumulation of osmoprotectants (Priya et al. 2019). Stress-related genes regulate the synthesis of secondary metabolites (e.g., flavonoids, terpenoids) and activate antioxidant defenses such as superoxide dismutase (SOD), catalase (CAT), ascorbate peroxidase (APX), and glutathione peroxidase (GPX) to mitigate oxidative damage under drought conditions (Hasanuzzaman et al. 2020). Similarly, aquaporins, by participating in water transport across membranes, maintain hydraulic conductivity, providing better adaptation during drought (Pawłowicz and Masajada 2019). Simultaneously, genes, such as MAPKs (Mitogen-activated protein kinases), were shown to act as signal transducers to activate stress-responsive pathways (Mahmood et al. 2020). Similarly, Pyrabactin Resistance 1-like (PYL-like) genes involved in ABA signaling were involved in stomata regulation and enhanced WUE under drought (Wang et al. 2025), while the transcription factors myeloblastosis (*MYB)* and *WRKY* are involved in the osmotic stress response and drought-induced oxidative stress defense (Price et al. 2022). Recent studies suggest that the presence of small RNAs are involved in the regulation of drought-responsive genes, thus adding an extra layer of post-transcriptional defense towards water deficit (Li et al. 2024).

Combining data from multiple aspects of plant response at the physiological, morphological, metabolic and molecular level not only leads to the effective breeding of drought-resistant olive cultivars but also reveals the stress memory of plants (Nteve et al. 2024; Skodra et al. 2023), a mechanism that allows for faster and more effective activation of response mechanisms to a recurring stress (Lagiotis et al. 2023). Inducing plant resilience through stress memory requires an initial stress event (priming event).  Liu et al. 2022a, ). Priming processes that support stress memory development have also been identified in olive plants. Ben Abdallah et al. (2017),investigated the priming effect of drought on the drought-sensitive "Chétoui" olive cultivar in Tunisia, showing that pre-exposure to drought stress led to higher stress tolerance. In a later study, the same group used salt priming to induce drought stress memory in "Chétoui" olives, which improved the plants' physiological and biochemical responses under drought (Ben Abdallah et al. 2022).

The present study aims to provide critical insights into the drought and priming-induced responses of two Greek olive cultivars, "Lefkolia Serron" and "Chondrolia Chalkidikis" which are mainly cultivated in Northern Greece and are particularly recognized for the quality characteristics of both their fruit and olive oil. This research focuses on two principal objectives: (i) to dissect the distinct physiological, morphological, metabolic, and gene expression responses of these olive cultivars under drought stress, and ii) to assess their ability to acclimate through cis-priming. The findings will help to understand the drought-tolerance mechanisms of olive trees, optimize breeding selection in resistant cultivars, and identify naturally resistant genotypes to drought. These strategies are essential to ensure the resilience and sustainability of olive cultivation amidst the intensifying challenges of climate change.

## Material and methods

### Plant material and growth conditions

Τwo-year-old trees of the cultivars “Lefkolia Serron” (LS) and “Chondrolia Chalkidikis” (CC), were used in this study. The experiment was performed in controlled greenhouse conditions at the Farm of Aristotle University of Thessaloniki, from June to October 2020. The average temperature ranged from 19.1 to 27.1 °C, and the average relative humidity ranged from 44 and 79%. A total of 90 plants were used, with an equal sample size of 45 plants per cultivar. The plants were grown in 3L pots, filled with 4:1 (v/v) potting soil and perlite.

### Experimental design and drought stress treatments

**Fig. 1:**
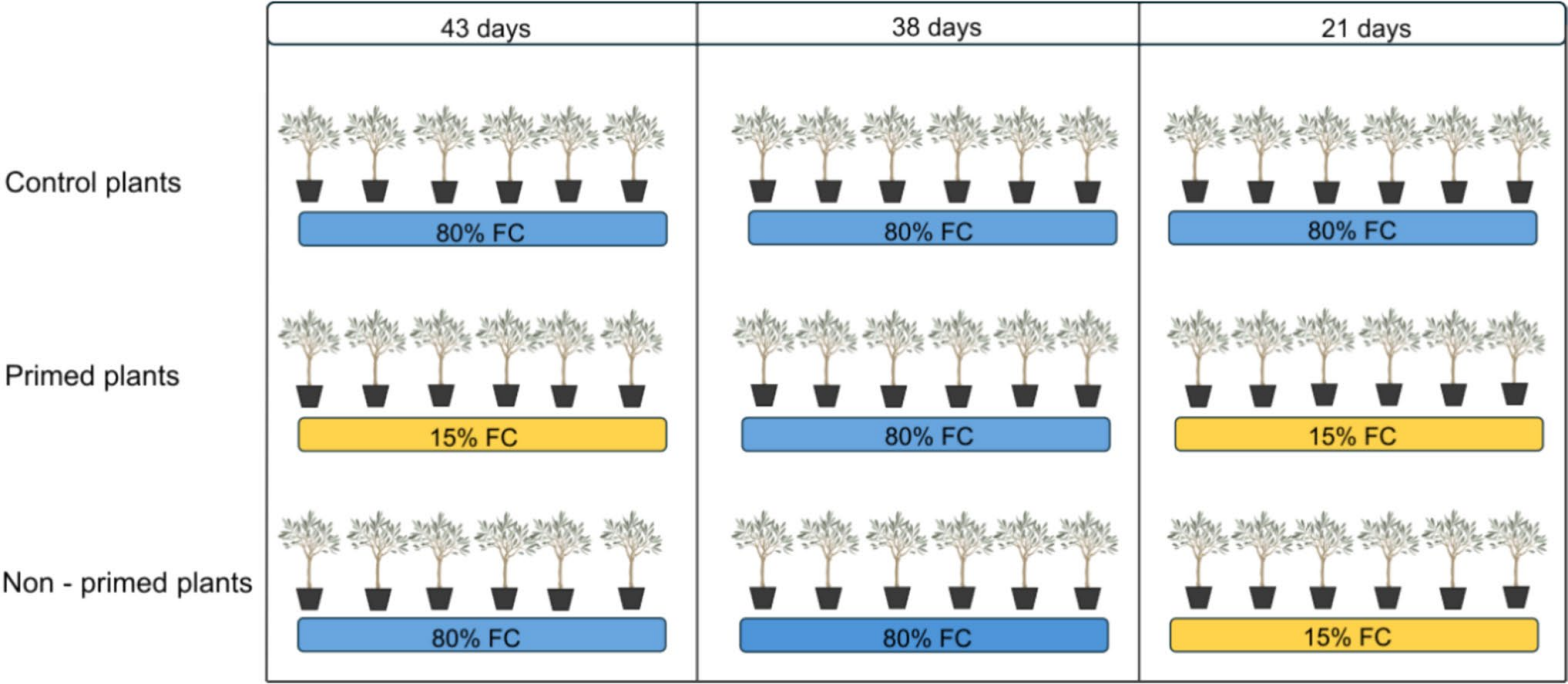
The experimental design for drought stress and priming. The design included three irrigation treatments (control-primed and non-primed) with 15 plants per treatment for both cultivars, LS and CC. All plants were irrigated based on field capacity (FC). Control plants were maintained at 80% FC throughout the experiment (102 days), representing optimal water supply. Primed plants were exposed to a long-term drought priming event at 15% FC for 43 days, followed by rehydration at 80% FC for 38 days, and then subjected to a second drought cycle at 15% FC for 21 days. Non-primed plants received optimal irrigation (80% FC) for 81 days but were exposed to a single drought episode at 15% FC for 21 days

The experiment consisted of two drought periods, with an intermediate rehydration phase, as a recovery period, to simulate real-environment changes in the water supply. The first period of drought was used as the priming event. Plants were grown in a completely randomized (CRD) split-plot design with three treatments and 15 plants (biological replicates) per treatment. The treatments included different irrigation regimes: (i) control plants (C) with constant and adequate water supply (80% Field Capacity, FC); (ii) primed plants (P), exposed to the priming event during the first drought cycle (15% FC), followed by a rehydration phase (80% FC), and then subjected to the second drought cycle (15% FC); and (iii) non-primed plants (NP), grown under optimal irrigation conditions (80% FC) during the first two phases of the experiment, but were then exposed to a single drought cycle (15% FC) in the third phase of the experiment (Fig. 1). The experimental design was adapted from Ben Abdallah et al. (2017) and relevant literature (Hilker et al. 2016; Turgut-Kara et al. 2020). The priming event of 43 days, combined with the significantly reduced water regime, could be considered as a long-term drought priming. Water was applied gravimetrically, and pot target weights were maintained by regular weighing and re-watering approximately every 2 days throughout the four months of the experiment. The amount of water added to each pot was calculated based on the target weight of the pot (PT), with the following equation:

1 $$ {\text{PT }} = {\text{ PD }} + {\text{ MW }} + {\text{ FC }} \times \, \left( {{\text{PW }} - {\text{ PD}}} \right) $$

where PD and PW were the dry and wet weights of the pot, including the substrate, respectively, measured before transplantation and target weights were adjusted for the growing biomass of each genotype (MW) using regular harvests of the above-ground biomass from plants growing in the same conditions (Stavridou et al. 2019). Leaf samples were collected at three time-points, corresponding to changes in the water regime during the experimental period, and stored at − 80 °C for further analysis.

### Physiological parameters

Physiological parameters, including relative chlorophyll content, CO_2_ assimilation rate (*A*), transpiration (*E*), stomatal conductance (*gs*) and chlorophyll *a* fluorescence (*Fv/Fm*) were evaluated. To capture the changes in plant water status throughout the experimental period, measurements were conducted on six biological replicates per treatment and at one time-point, within each of the three distinct phases of the study as described in Table 1.

**Table 1:** Time points and corresponding days of the experiment related to physiological measurements

Time point	Experimental day	Description
T1	43	End of the long-term priming event
T2	44	24 h after re-watering the primed plants
T3	102	End of the experiment, in which primed and non-primed plants were under drought

### Relative chlorophyll content

The relative chlorophyll content (RCC) was determined according to Stavridou et al. (2017). Measurements were performed on the adaxial leaf surface of three fully expanded leaves per plant, located on the upper half of the plant, using the CCM-200 plus Chlorophyll Content Meter (Opti-Sciences, Inc., Hudson, NH, USA). Each measurement was an average of three independent measurements per leaf.

### Chlorophyll *α* fluorescence

The maximum quantum yield of Photosystem II (*Fv*/*Fm*), serving as a photodamage indicator of PSII, was measured on the adaxial leaf surface of the youngest fully expanded leaf using the OS30p + Chlorophyll Fluorometer (Opti-Sciences, Inc., Hudson, NH, USA) (Stavridou et al. 2017). Leaves were dark adapted for 30 min using light exclusion clips. Chlorophyll *α* fluorescence parameters, such as *F*_*O*_ (minimal fluorescence) and *Fm* (maximum chlorophyll fluorescence), were recorded, calculating the maximum quantum yield efficiency of PSII as following: *Fv/Fm* = (*Fm -Fo)/ Fm*.

### Gas exchange measurements

Gas exchange measurements of *A*, *E*, *gs*, were recorded in situ from 8:00 to 12.00 h on fully expanded leaves of each plant, using the portable LCi T compact photosynthesis system gas exchange analyzer (ADC BioScientific Ltd, Herts, England) (Skodra et al. 2023). Intrinsic Water Use Efficiency (iWUE) was assessed to evaluate the plant’s ability to balance water loss and carbon assimilation under drought conditions and was calculated as the ratio of the photosynthetic rate (*A*) to the stomatal conductance (*gs*) using the formula: iWUE = *A*/*gs*.

### Morphological measurements

At the end of the experiment (Τ3), several morphological parameters were assessed, to evaluate plant performance under drought stress and the effect of the number of drought cycles (two vs one). Traits, such as shoot, and root length (cm) were measured on six plants per treatment. Regarding dry matter accumulation, six plants per treatment were harvested. The plants were separated into root and shoot parts, and their fresh weights were measured. The dry weights were measured after drying the root and shoot parts at 70 °C for 72 h. Biomass allocation was assessed using the root-to-shoot ratio calculated on six plants per treatment as R/S = Root Dry Weight / Shoot Dry Weight. To determine the moisture status in different plant tissues, the water content of the shoot (WCS), and root (WCR) was calculated as: WC = [(Fresh weight – Dry weight) / Fresh weight)] × 100.

### Metabolomic profiling

Metabolomic analysis of primary metabolites was conducted on olive leaves, to investigate the changes in their profiles. Leaves from three plants per treatment were collected at each of the three experimental time points (T1, T2, and T3), as summarized in Table 1 and described in Karagiannis et al. (2021) (Supplementary Material, Online Resource 1). The GC–MS analysis was carried out with a Perkin Elmer Clarus™ SQ 8 (Waltham, MA, USA), as described in Skodra et al. (2023). The peak areas of the identified compounds were quantified using the OpenChrom software, for reliable integration and analysis of chromatographic data. Compounds were determined using standards or either the NIST11 or GOLM metabolome databases (Michailidis et al. 2019). The metabolites were expressed as the relative abundance of adonitol and are provided in Supplementary Material, Online Resource 5.

### Gene expression patterns

Gene expression analysis was performed to complement and validate physiological and metabolomic results. Eighteen leaf samples, consisting of three biological replicates for the three treatments, from both cultivars, were sampled during the final time-point (T3) and used to determine the expression profiles of specific drought-responsive genes. Total RNA was extracted using the NucleoSpin® RNA Plant Kit (Macherey–Nagel, Germany) according to the manufacturer's instructions. RNA concentration and purity were assessed with the Quawell UV Vis spectrophotometer (Q5000 US), and RNA integrity was also confirmed on 2% agarose gel. The samples were then subjected to cDNA synthesis using the Superscript II reverse transcriptase kit (Invitrogen, USA), with 500 ng of total RNA in each reaction, according to the manufacturer's instructions. Real-time quantitative reverse transcriptase PCR (qRT-PCR) was conducted on the Applied Biosystems StepOnePlus™ Real time PCR system, using the comparative Ct method. Three biological cDNA samples with two technical replicates per sample were used to determine the accuracy and reproducibility of the reactions (Supplementary Material, Online Resource 2). Data were analyzed using the ΔΔCt method and were expressed as 2^*−ΔΔCt*^ (Livak and Schmittgen 2001). Primers were designed to target key gene families involved in drought stress responses listed in Supplementary Material, Online Resource 2. Gene sequences were obtained from the Phytozome database, and PrimerQuest was used to design the primers. Additionally, the specificity of the designed primers was validated using BLAST (http://blast.ncbi.nlm.nih.gov/Blast.cgi). The gene Elongation Factor 1-alpha (*EF1A*) was used as the reference gene for normalization in the gene expression analysis (Ray and Johnson 2014).

#### Statistical analysis

For the comparison of the two cultivars, among the three treatments and for the interaction cultivar × treatment at the same time point, means and standard deviations were calculated for all physiological, morphological, metabolomic, and gene expression data. Prior to analysis, metabolomic datasets were log2-transformed. Two-way ANOVAs were performed to test for statistically significant differences, followed by Tukey's Honestly Significant Difference (HSD) post hoc test for mean separation. Percentage changes in physiological parameters of primed plants were calculated using the formula:

2 $$\frac{(New value-Old value)}{Old value}\times 100$$

for comparisons after re-watering (T1 *vs* T2) and between the two drought cycles (T1 *vs* T3). Hierarchical clustering was performed on the metabolite abundance data using Euclidean distance and the average linkage method to identify metabolite groups. A heatmap based on average correlation was subsequently used to visualize the pairwise relationships and co-regulation patterns among these metabolites. Principal Component Analysis (PCA) was additionally performed to interpret gene expression data. The significance level in all statistical tests was predetermined at *a* = 0.05 (*p*-value ≤ 0.05). The analyses and visualizations were performed in OriginPro, GraphPad, SRplot and ClustVIS software.

## Results and discussion

In the present study, two Greek-oriented olive cultivars, "Lefkolia Serron" (LS) and "Chondrolia Chalkidikis" (CC), were subjected to drought stress and long-term priming to investigate their morpho-physiological, biochemical and molecular responses and determine whether prior stress exposure enhances drought tolerance.

### Priming enhances drought acclimation through morpho-physiological adjustments

Percentage differences in physiological traits between LS and CC primed plants across phases (T1 vs. T2, T1 vs. T3) revealed genotype-specific responses (Table 2). CC exhibited a notably higher recovery in photosynthetic rate (*A*), transpiration rate (*E*), and stomatal conductance (*gs*) compared to LS after re-watering (T1 vs T2). However, after two cycles of drought, LS showed a greater increase in iWUE and a more conservative water management strategy. Although *gs* was higher in LS, this did not result in a proportional increase in *E*, unlike in CC, where higher *gs* was associated with greater water loss.

**Table 2:** Percentage changes (%) in physiological parameters of primed plants between LS and CC based on two comparison intervals: T1 versus T2 and T1 versus T3. T1 corresponds to the end of the priming event (day 43), T2 to 24 h after re-watering (day 44), and T3 to the end of the experiment, during which primed plants were under drought stress for a second time (day 102)

Cultivars	LS	CC	LS	CC
Physiological parameters	T1 vs. T2 (%)		T1 vs. T3 (%)	
RCC	0.3	− 2.05*	0.81	16.3
*F*_v_/*F*_m_	0.12	0.5	− 0.24	− 6.2
*A*	19.6	115.6	87.8	103.9
*E*	38.3	52.4	9.72	39.7
*g*_s_	50	100	75	66.6
iWUE	− 1.95	− 3.79	1.9	0.45

^*^Positive values indicate an increase, whereas negative values indicate a decrease

### Genotype dependent- changes in chlorophyll content and PSII efficiency

**Fig. 2:**
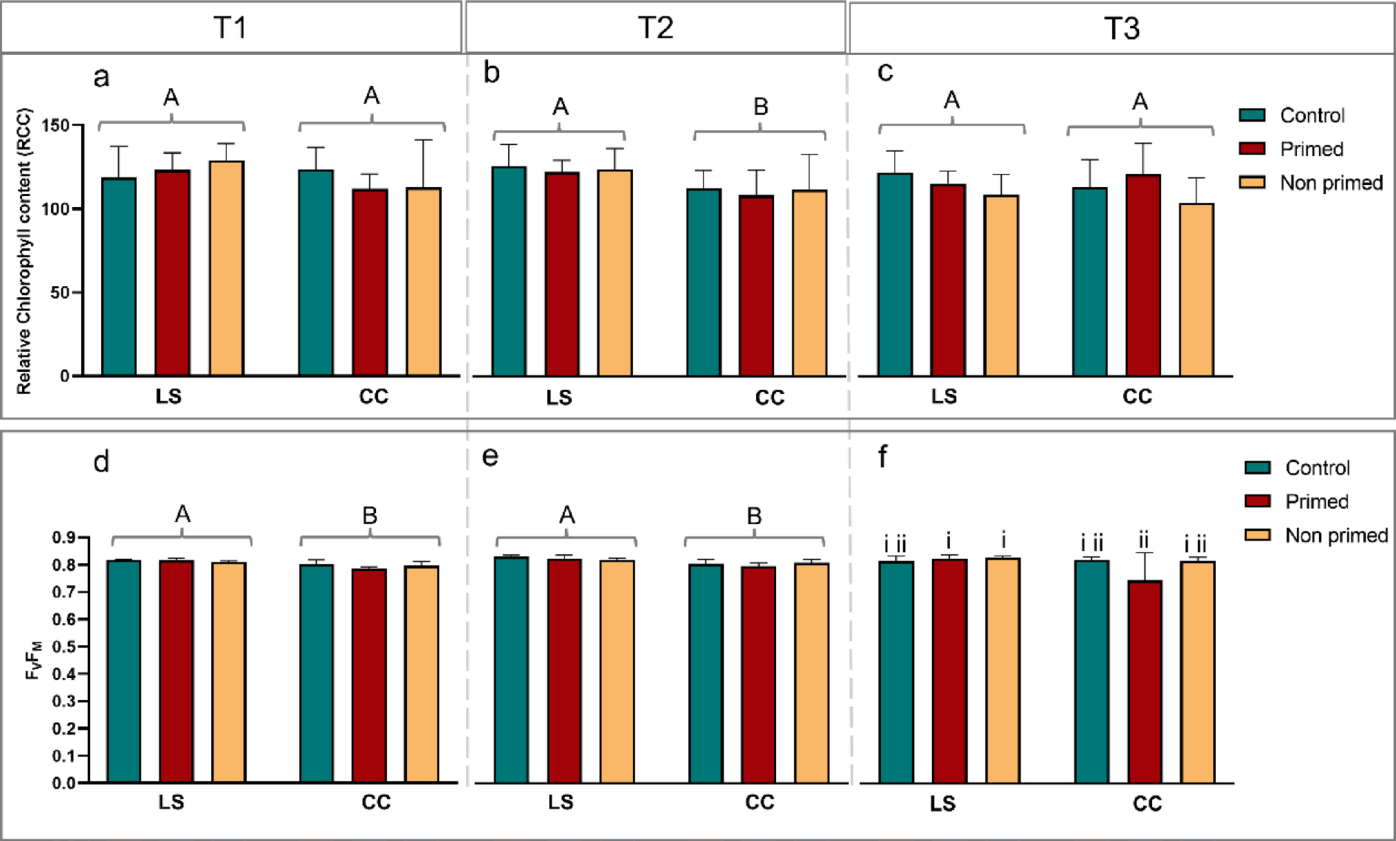
Relative Chlorophyll content (A-B-C) and Chlorophyll* a* fluorescence (*Fv/Fm*) (D-E–F) of cultivars LS and CC among control, primed and non-primed conditions at three time points (T1, T2, T3). The bars represent mean values ± standard error. Different letters denote statistical significance (*p*-value < 0.05) for each time point according to Tukey's multiple post hoc tests. Different capital letters indicate significant cultivar differences; different lowercase letters indicate significant treatment differences within each cultivar; different Roman numerals (I, II, III) indicate significant genotype x treatment interaction; absence of letters/numerals indicates no significant effect

After 43 days of drought (T1 cycle), the Relative Chlorophyll Content (RCC) showed no significant differences between treatments or cultivars (Fig. 2a). Similarly, Boussadia et al. (2023) observed no reduction in the RCC after a period of 45 days-stress, in most of the Tunisian olive cultivars. However here, after 24 h of rehydration (T2), RCC differed significantly between the two cultivars (Fig. 2b). LS, primed plants showed a slight increase of 0.3%, suggesting limited recovery (Table 2). In contrast, primed plants of CC showed a 2.05% decrease, indicating a slower or potentially ineffective response following rewatering (Table 2). At T3 (Fig. 2c), RCC was higher in primed plants of both cultivars compared to non-primed plants, though not statistically significant. CC showed a marked 16.3% increase, while LS increased by just 0.81%. This aligns with (Ben Abdallah et al. 2017) who reported increased RCC in primed olives under moderate stress. The enhanced RCC in CC suggests that priming may promote chlorophyll retention, possibly for the protection of photosynthetic machinery (Sintaha et al. 2022).

Chlorophyll *α* fluorescence reveals the functional status of Photosystem II (PSII), and the extent of photodamage under excess light (Petridis et al. 2012). However, PSII has a higher relative tolerance to water deficit compared to PSI and negative effects only appear in severe drought conditions (Lauriano et al. 2006). In our study, 43 days of drought (Fig. 2d) and re-watering (Fig. 2e) did not significantly affect (*Fv/Fm*) across treatments, however LS showed significantly higher values compared to CC, indicating less PSII photodegradation. Similarly, Ben Abdallah et al. 2017, 2018) reported that the *Fv/Fm* in drought-stressed "Chétoui" olive plants remained unaffected suggesting preserved PSII efficiency. Although treatment effects were not significant, cultivar differences highlight the importance of genetic background in drought stress resilience. At T3 (Fig. 2f), after the final 21-days-drought significant differences were observed. LS primed plants, showed only a 0.24% decrease in *Fv/Fm* (Table 2), suggesting enhanced photoprotection possibly due to priming. Conversely, CC primed plants showed a 6.23% decline, indicating limited protective mechanisms. These findings suggest that PSII in LS primed plants is more resilient across fluctuating water regimes, likely due to photoprotection, antioxidant mechanisms and rapid repair processes, allowing recovery without significant changes in photosynthetic pigments (Petridis et al. 2012). On the other hand, the lower *Fv/Fm* in CC, indicates a rather photosensitive PSII system (Jat et al. 2024). Nevertheless, a slight decrease in *Fv/Fm* under drought stress allows olive plants to maintain electron transport and avoid severe photodamage (Guerfel et al. 2009).

### Stomatal regulation contributes to enhanced photosynthetic performance

**Fig. 3:**
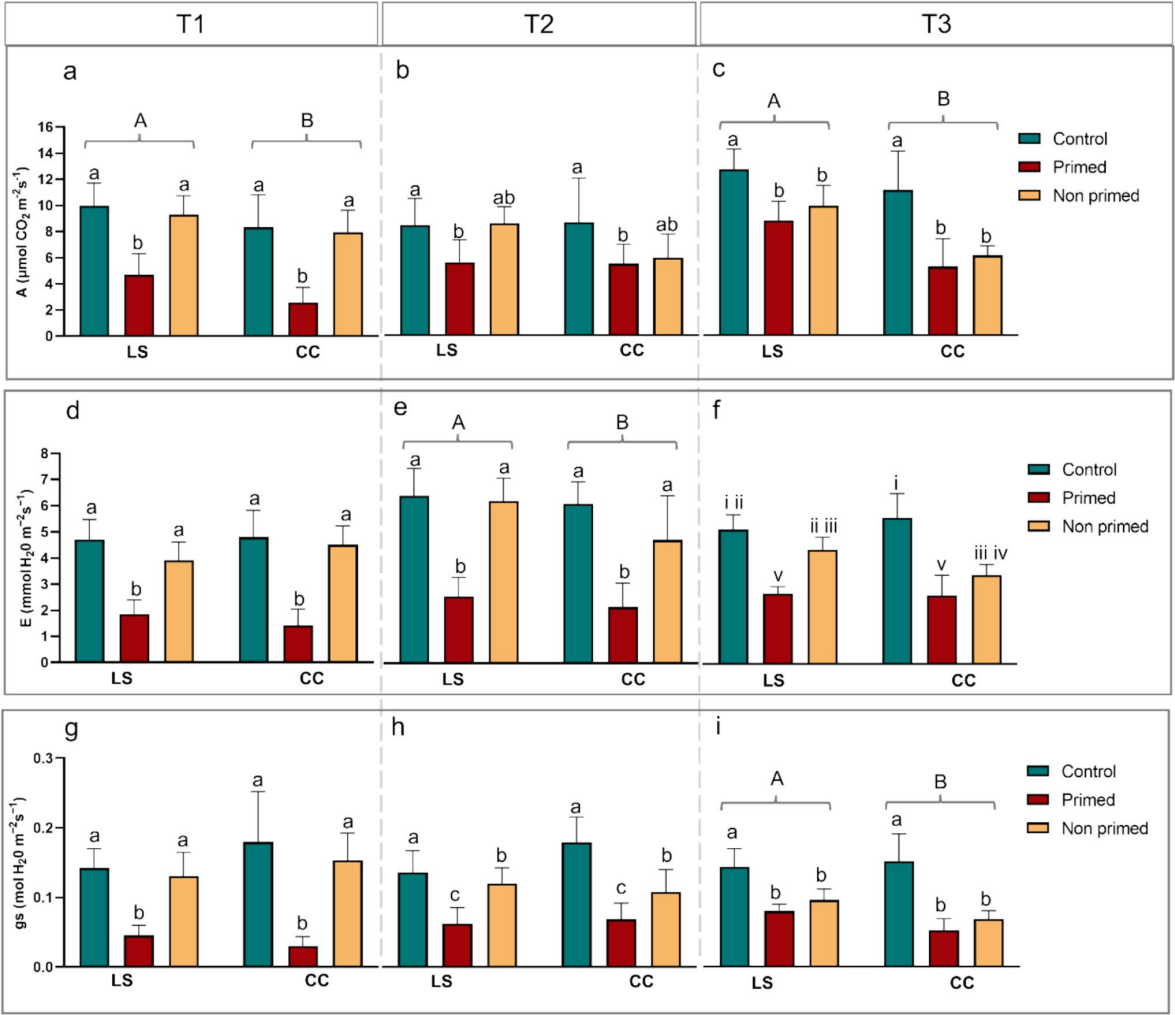
Photosynthetic rate, *A* (A-B-C), transpiration rate, *E* (D-E–F) and stomatal conductance *gs* (G-H-I) of cultivars LS and CC among control, primed and non-primed conditions at three time points (T1, T2, T3). The bars represent mean ± standard error. For each time point, different letters denote statistical significance (*p*-value < 0.05) according to Tukey’s multiple post hoc tests. Different capital letters indicate significant cultivar differences; different lowercase letters indicate significant treatment differences within each cultivar; different Roman numerals (I, II, III) indicate significant genotype x treatment interaction; absence of letters/numerals indicates no significant effect

During the 43-day drought-priming period, *A*, *E* and *gs* were significantly reduced in primed plants of both cultivars, compared to well-watered control and non-primed plants (Fig. 3a, d, g). Notably, LS showed higher *A* than CC (Fig. 3a), whereas for *E* (Fig. 3d) and *gs* (Fig. 3g), both cultivars showed no statistical differences. Stomatal limitations typically reduce the photosynthetic rate, further affecting plant growth (Cernusak 2020), which could explain the observed reduction in *A* of primed plants. In olive trees under drought, similar findings confirm the reduction of these parameters (Ben Abdallah et al. 2017; Petridis et al. 2012). According to (Brito et al. 2018) the significant reduction of *gs* and *A* in drought-stressed plants is a form of acclimation mechanism for water conservation.

Crop performance depends not only on responses during a stress period, but also on those occurring before its onset and during recovery (Feller 2016). Here, after 24 h of re-watering, primed olive plants showed rapid recovery in gas exchange parameters; however, their values remained lower than those of the well-watered plants (Fig. 3b, e, h). Comparisons between T1 vs T2 showed that CC exhibited a remarkable recovery in *A* (115.6%), *gs* (100%) and *E* (52.4%) compared to LS, with increases of 19.6%, 50%, and 38.3%, respectively (Table 2). This rapid recovery in CC suggests that its photosynthetic system could better respond to rehydration and that drought stress did not cause irreversible damage (Yang et al. 2023). This trend may indicate a partial restoration of water uptake and leaf cooling capacity through evapotranspiration (Sintaha et al. 2022).

During the second drought phase (T3), LS exhibited higher *A* than CC (Fig. 3c). Within cultivars, primed and non-primed plants showed similar rates, both lower than control plants. Interestingly, primed plants from both cultivars exhibited improved gas exchange rates during the second drought exposure (T3) compared to the priming phase (T1) (Fig. 3c, f, i). This highlights that priming enhanced physiological functionality after subsequent exposure to drought. Comparing the two drought cycles of primed plants, we observed an 87.8% increase in *A* in LS, whereas a notable 103.9% increase was found in CC (Table 2). Overall, primed plants maintained photosynthetic performance after the second exposure to drought, probably due to priming. Similar results were reported by Ben Abdallah et al. (2017) regarding primed olive plants; however, photosynthetic parameters of non-primed plants were negatively affected by drought likely due to complete lack of water, as opposed to the 15% water limited supply in our experiment.

Primed plants of both cultivars effectively regulated stomatal opening to reduce water loss through transpiration, compared to non-primed plants, subjected to one drought cycle. This performance suggests that prior exposure to drought may enhance water retention during subsequent stress by reducing water loss through stomata control. Similarly, Sintaha et al. (2022) found that soybean plants subjected to two drought cycles exhibited lower values of *E* and *gs*, compared to those that experienced only one drought cycle, supporting potential benefits of priming in enhancing drought tolerance. This suggests that pre-existing stress may induce physiological adjustments, so that in a recurring event, the regulation of stomata may improve water retention in the leaf while ensuring photosynthetic functionality. Based on differences in physiological responses, LS and CC follow different defense strategies likely due to their genetic profile. Generally, depending on water availability, LS adopts a more conservative management of water resources, while CC seems to follow more drastic changes, with greater sensitivity to water scarcity and more immediate recovery when water is available.

### Leaf iWUE enhancement as a key indicator of olive plant acclimation to water deficit

**Fig. 4:**
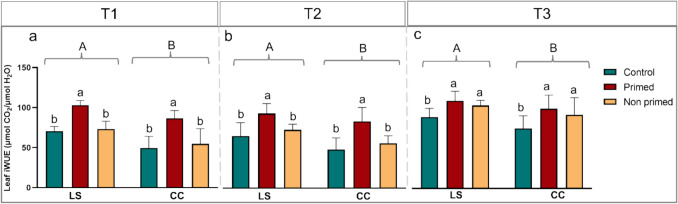
Leaf iWUE of cultivars LS and CC among control, primed and non-primed conditions at three time points (T1, T2, T3). The bars represent mean values ± standard error. For each time point, different letters denote statistical significance (*p*-value < 0.05) according to Tukey’s multiple post hoc tests. Different capital letters indicate significant cultivar differences; different lowercase letters indicate significant treatment differences within each cultivar; different Roman numerals (I, II, III) indicate significant genotype x treatment interaction; absence of letters/numerals indicates no significant effect

Leaf intrinsic WUE (iWUE) represents the physiological balance between the photosynthetic assimilation of CO_2_ and water loss through the stomatal pores. As shown in Fig. 4, during all time points (T1, T2 and T3), primed plants of both cultivars had significantly higher iWUE, compared to the control and non-primed plants, reaching a maximum value in T3. Furthermore, at all time-points, LS had higher iWUE compared to CC. WUE is an important adaptive trait that can improve crop productivity under conditions of water scarcity (Mashilo et al. 2017). The higher value of primed plants even after recovery suggests that priming improved stomatal regulation and ultimately optimized photosynthesis.

### Repeated drought episodes trigger acclimation responses in the roots of olive plants

At T3, the responses of LS and CC to different watering treatments were evaluated in terms of shoot and root traits, as summarized in Table 3 No significant differences were observed between cultivars; however, CC outperformed LS in all traits except shoot length. Regarding treatments, significant differences were detected in fresh and dry shoot weight, as well as in fresh root weight. Optimal irrigation allowed control plants to develop the highest values across all traits, followed by non-primed and primed plants. It is noteworthy that primed plants showed increased root length than non-primed plants. This difference can be attributed to adjustments in the root architecture caused by the different stress cycles (Calvo-Polanco et al. 2019). This aligns with previous findings, as in drought conditions, olive cultivars and other plant species tend to prioritize root development and plasticity over shoot growth (Nteve et al. 2024; Tadić et al. 2024). While stress reduced fresh and dry shoot weight in both primed and non-primed plants, only primed plants showed a significant reduction in fresh root weight. This may reflect the reduced moisture content of the roots in primed plants, which experienced two drought cycles. Such responses likely include the synthesis of osmo-protective and antioxidant compounds (Fleta-Soriano and Munné-Bosch 2016). Although these mechanisms enhance tolerance in olive plants, they are energetically demanding and may compromise root biomass production.

**Table 3:** Morphological and biomass measurements for shoot and root of cultivars LS and CC under control, primed and non-primed conditions

	Shoot			Root		
	Length (cm)	Fresh weight (gr)	Dry weight (gr)	Length (cm)	Fresh weight (gr)	Dry weight (gr)
*Cultivars*						
LS	83.83 ± 2.6 A	41.22 ± 1.8 A	22.50 ± 1.3 A	24.06 ± 1.5 A	8.17 ± 0.4 A	4.94 ± 0.2 A
CC	82.22 ± 3.1 A	43.22 ± 3.4 A	23.28 ± 2.0 A	29.94 ± 2.8 A	8.94 ± 0.5 A	5.22 ± 0.3 A
*p*-value	0.697	0.516	0.735	0.086	0.153	0.505
*Treatments*						
Control	84.67 ± 2.5 a	51.33 ± 3.1 a	27.33 ± 2.0 a	29.08 ± 3.6 a	9.67 ± 0.6 a	5.33 ± 0.3 a
Primed	78.67 ± 3.7 a	33.42 ± 1.1 b	18.67 ± 0.9 b	27.33 ± 2.0 a	7.08 ± 0.3 b	4.67 ± 0.3 a
Non-primed	85.75 ± 3.9 a	41.92 ± 2.9 b	22.67 ± 2.3 ab	24.58 ± 2.8 a	8.92 ± 0.4 a	5.25 ± 0.3 a
*p*-value	0.330	< 0.001	0.015	0.544	0.001	0.367

Values represent means ± standard error. Different letters denote statistical significance (*p*-value < 0.05) according to Tukey’s multiple post hoc tests. Capital letters refer to differences between cultivars, and lower-case letters refer to differences among treatments per cultivar

Table 4 presents the root-to-shoot ratio (R/S) and the water content (WC) of shoots and roots for LS and CC across control, primed and non-primed plants. R/S ratio showed no significant cultivar differences, whereas significant differences were observed among treatments. Primed plants exhibited the highest R/S ratio, followed by non-primed plants, while control plants had the lowest ratio. A higher R/S ratio indicates greater allocation of resources to root growth and less to shoot growth, helping to access water in deeper soil (Nteve et al. 2024; Sadhukhan et al. 2024). This energy reallocation, where the plant invests in structural reinforcement instead of unnecessary vegetative growth, is a form of strengthening resilience through priming, which has been found in other tree species such as Citrus sp. (Scialò et al. 2025). This adjustment can be considered as a water stress tolerance strategy to increase water intake under stress conditions.

**Table 4:** Root-to-shoot ratio (R/S) and shoot and root water content (WC) of cultivars LS and CC under control, primed and non-primed conditions

	R/S ratio	Shoot WC (%)	Root WC (%)
*Cultivars*			
LS	0.22673 A	44.5 A	38.7 A
CC	0.23558 A	44.7 A	39.6 A
*p*-value	0.56767	0.97328	0.88056
*Treatments*			
Control	0.20013 b	45.4 a	42.7 a
Primed	0.25107 a	43.5 a	35.3 a
Non-primed	0.24227 ab	44.9 a	39.3 a
*p*-value	0.024	0.958	0.594

Values represent means ± standard error. Different letters denote statistical significance (*p*-value < 0.05) according to Tukey’s multiple post hoc tests. Capital letters refer to differences among cultivars and lower-case letters refer to differences among treatments per cultivar

WC is a valuable indicator for assessing plant water status and drought tolerance (Boussadia et al. 2023). For Shoot and Root WC, no significant differences were recorded between cultivars or treatments. Generally, WC decreases in drought conditions (Gholami et al. 2022) yet drought-tolerant cultivars maintain high WC values compared to the drought-sensitive ones (Karimi et al. 2018). The longer roots and the slightly higher root WC of CC, although not statistically significant, suggest a more efficient water uptake strategy under stressful conditions.

### Priming-dependent versus conserved metabolic responses in CC and LS olive plants

Metabolomic analysis at T1 and T2 (Supplementary Material, Online Resource 3) shows the relative abundance of primary metabolites in leaf samples across control, primed and non-primed conditions, for LS and CC. At both time points, more than 20 metabolites were identified, including sugars, sugar alcohols, organic acids, alcohols and other compounds. During T1, primed plants in both cultivars had increased stress-related metabolites, such as osmo-protectants and organic acids, reflecting adaptive responses towards stress (Supplementary Material, Online Resource 6). After re-watering, LS plants returned to normal metabolism with fewer indications of stress metabolites, whereas CC accumulated energy-storage compounds, indicating different response strategies (Supplementary Material, Online Resource 7). These results reveal genotype-specific metabolic adjustments to drought stress and recovery (Obata and Fernie 2012).

**Fig. 5:**
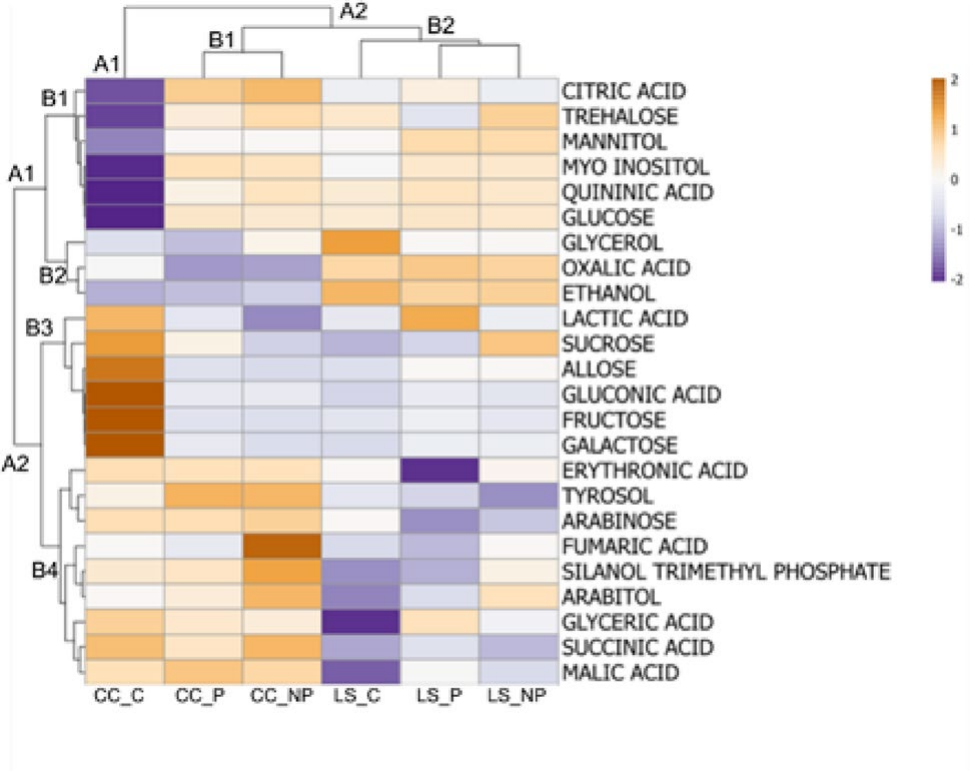
Heatmap of primary metabolite changes in leaf samples collected at T3, (day 102), representing LS and CC among control (C), primed (P), and non-primed (NP) conditions. The heatmap illustrates the log₂-transformed relative abundance (1 mg/mL adonitol as internal standard) of primary metabolites, with blue indicating decreased metabolite levels and orange indicating increased metabolite levels across the different treatments. Hierarchical clustering is shown for both metabolites (left) and experimental groups (top)

Figure 5 presents the metabolic profiles of olive leaf samples at T3, revealing different metabolic patterns among both cultivars and applied treatments. In total, 24 metabolites were identified, including sugars and sugar alcohols, organic acids, alcohols and one other compound. Metabolites were clustered into two main groups (A1-A2), each with sub-clusters B1 and B2. Treatments were also divided into two main clusters: A1, including control plants of CC, and A2, including all other treatments. Within A2, primed and non-primed plants of CC were clustered into subgroup B1. Likewise, B2 included all treatments of LS plants. Among the 24 metabolites,13 showed significant differences between LS and CC, 7 among treatments, and 6 for cultivar x treatment interaction (Supplementary Material, Online Resource 8 & Online Resource 9).

In LS, metabolite accumulation showed similar patterns in all treatments during Τ3, suggesting a stable biochemical adjustment under different water regimes. Primed plants of LS displayed an increase in lactic and glyceric acid and a decrease in erythronic acid. Lactic acid is often linked to fermentative metabolism, maintaining cellular pH and energy balance under water deficit. Glyceric acid facilitates glycolysis and energy production pathways (Petridis et al. 2012). Repeated drought cycles expose olive trees to progressively stronger metabolic adjustments involving organic acids (Dias et al. 2021). Although organic acids have been extensively studied, their role in stress resistance has not yet been well documented (Panchal et al. 2021). Regarding trehalose, which typically increases under drought conditions (Shao et al. 2022), our study showed that control and non-primed plants of LS increased trehalose levels compared to primed plants. Regarding the non-primed plants of LS, elevated levels of arabitol, and sucrose were detected, metabolites associated with osmotic adjustment and energy supply under drought. Arabitol, a polyol, stabilizes cell structures alongside maintaining osmotic equilibrium under drought conditions (Bacelar et al. 2007; Kumar et al. 2021). Accumulation of sugars, such as sucrose, glucose, galactose, maltose, lactose, raffinose and fructose, is consistently driven by drought, altering the source-sink relationship in plants (Kaur et al. 2021). Such osmolytes, and other compatible solutes, play a major role in preserving cellular structures under water stress (Sharma et al. 2019). In general, LS showed uniformity in the metabolite profile, suggesting resilience to drought stress through maintaining homeostasis under different water regimes.

In CC, control plants showed a distinct metabolic profile with significant variations in specific metabolites, compared to primed and non-primed plants, which exhibited similar metabolic profiles between them. Myo-inositol, citric acid, glucose, quininic acid and trehalose were increased in primed and non-primed plants compared to the reduction observed in control plants. Primed plants often exhibit more stable metabolic profiles likely due to 'stress imprinting', which allows them to better regulate osmoprotectants during subsequent stress events (Dias et al. 2021). Non-primed plants, although less 'experienced' than primed plants, may exhibit intermediate metabolic responses as they begin to adjust to drought conditions (Schwachtje et al. 2019). Sugars such as fructose, allose, sucrose, galactose, and amino acids like gluconic and lactic acid showed higher concentrations in control plants, compared to primed and non-primed plants. This significant increase in primary metabolites could reflect a metabolic response to well-watered conditions, allocating more resources to processes associated with normal plant growth (Kumar et al. 2021). This variation in the metabolic activity among treatments in CC highlights the importance of (i) water availability on olive trees and their drought acclimation responses, and (ii) genetic profile in determining the plant’s response to drought stress.

### Drought priming ‘restores’ gene expression in olive plants

**Fig. 6:**
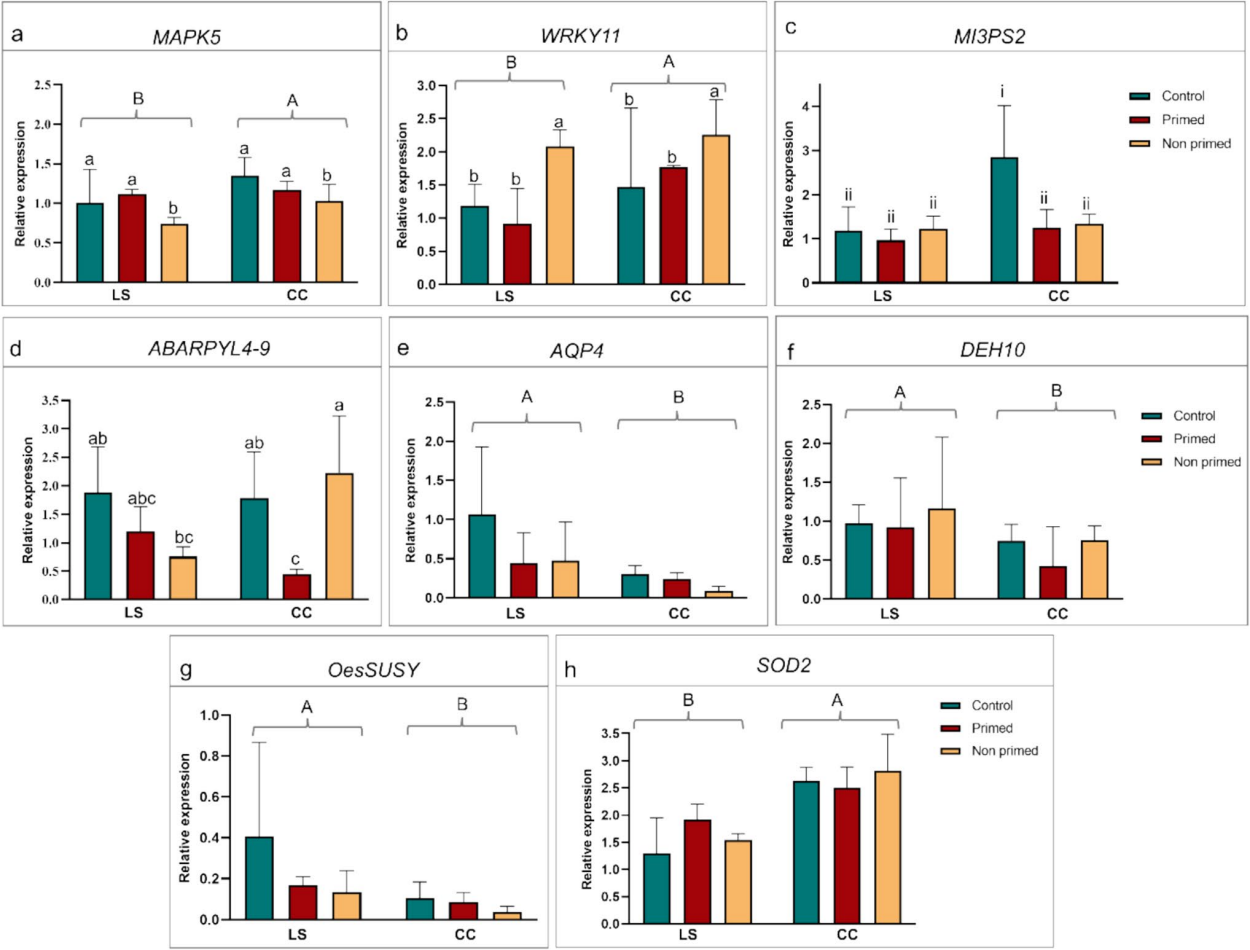
Relative gene expression analysis of drought-related genes *MAPK5*, *WRKY11*, *MI3PS2 ABARPYL4-9*, *AQP4*, *DEH10*, *OesSUSY* & *SOD2* of cultivars LS and CC among control, primed and non-primed conditions at the final day of the experiment (T3). The bars represent the means and standard errors of three biological replicates. Different letters denote statistical significance (*p*-value < 0.05) according to Tukey's multiple post hoc tests. Different capital letters indicate significant cultivar differences; different lowercase letters indicate significant treatment differences within each cultivar; different Roman numerals (I, II, III) indicate significant genotype x treatment interaction; absence of letters/numerals indicates no significant effect

To investigate the effect of drought priming on the molecular response of the two olive cultivars, we assessed the expression of 11 drought-responsive genes at the final time-point (T3) using RT-qPCR. The expression of eight drought-related genes (*MAPK5*, *WRKY11*, *MI3PS2 ABARPYL4-9*, *AQP4*, *DEH10*, *OesSUSY* & *SOD2*) showed statistical differences following the drought treatments (Fig. 6), whereas *ProDH*, *STOMCTDEF10* and *STI1* showed no statistically significant changes (Supplementary Material, Online Resource 4).

Particularly, the *MAPK5* expression was higher in CC than in LS (Fig. 6a), with primed plants of both cultivars showing higher expression compared to non-primed plants. As a key component of extracellular signal transduction, *MAPK5* may function as a molecular switch mediating stress adaptation (Lin et al. 2021). Increased *MAPK5* expression in primed plants, as in control plants, support the idea that drought priming helps maintain a transcriptional state similar to non-stress conditions.

Moreover, the expression of the transcription factor, *WRKY11* followed a similar pattern, with higher expression in CC than LS (Fig. 6b). Non-primed plants exhibited higher *WRKY11* expression, possibly indicating greater stress sensitivity, while primed plants maintained moderate levels closer to control, supporting a more efficient response to water deficit. The *WRKY* family positively regulates a variety of physiological processes and enhances abiotic stress responses in various woody plants (He et al. 2012; Yan et al. 2014).

Τhe *MI3PS2* involved in the synthesis of the drought-associated osmoprotectant myo-inositol phosphate exhibited significant cultivar x treatment interaction (Fig. 6c). In CC control plants, *MI3PS2* was upregulated, while the metabolite myo-inositol was reduced (Fig. 5), possibly reflecting incomplete metabolite synthesis at T3. The higher expression levels of *MI3PS2* in CC may reflect an early-stage response, as the synthesis of the corresponding metabolite had not yet been completed at that specific time point (Jia et al. 2019).

Regarding the *ABARPYL4-9*, a key component in Abscisic Acid (ABA) signaling pathway, showed treatment-specific differences (Fig. 6d). Generally, PYR/PYL/RCAR proteins were shown to enhance drought tolerance in many plant species, (Pizzio et al. 2013). In LS, non-primed plants had the lowest expression, whereas non-primed plants of CC exhibited the highest expression. These patterns suggested distinct genotypic ABA responses to water deficit (Todaka et al. 2019) with LS maintaining more stable ABA regulation than the more reactive CC, highlighting the distinct drought adaptation strategies.

The aquaporin *AQP4* expression (Fig. 6e), was higher in LS compared to CC, indicating a clear cultivar-specific response. Aquaporins (AQPs*)* facilitate water and solute transport in the water column along with uncharged solutes across membranes (Moshelion et al. 2015). The expression of *AQP4*, for primed and non-primed plants of both cultivars, was affected by drought, similarly to the work of (Faize et al. 2020), where the transcript levels of aquaporins were differentially downregulated in leaves, roots and twigs of olive trees after drought treatment.

Similarly, the dehydrin, *DEH10*, known for its role in osmoprotection (Sun et al. 2021) showed higher expression in LS compared to CC (Fig. 6f). Even though no significant differences were observed among treatments, non-primed plants in both cultivars showed a slight increase in *DEH10*, suggesting that olive plants experiencing drought for the first time induced a stronger dehydrin response. Sezer (2024) showed that specific dehydrin genes exhibited over sixfold higher levels upon drought, indicating their critical role in olive tree stress response. Interestingly, primed plants did not induce dehydrin expression suggesting that they had either adjusted their stress response or required fewer dehydrins after priming.

*OesSUSY* expression was also higher in LS compared to CC (Fig. 6g), aligning with the notion that LS exhibits stronger drought tolerance via enhanced carbohydrate metabolism. This expression pattern supports LS’s tolerance, as it has been shown that cytoplasmic sucrose synthesis was higher under drought conditions and there was differential expression in resistant and susceptible varieties (Thomas and Beena 2021). However, similarly to *MI3PS2* and myo-inositol synthesis, post-transcriptional and metabolic regulatory mechanisms seem to influence sucrose synthesis and degradation, since sucrose levels were only higher in the control plants of CC and non-primed plants of LS (Fig. 5). Generally, sugar metabolism is involved in the response and adaptation of olive trees to drought, including changes in the levels of simple sugars such as sucrose and glucose (Brito et al. 2019). Despite transcriptional upregulation, metabolite levels suggested post-transcriptional regulation, potentially due to enzyme activation or sugar partitioning (Ren et al. 2023).

The opposite effect was observed in *SOD2*, exhibiting a higher expression in CC compared to LS (Fig. 6h). Generally, water stress and salinity were shown to induce the total *SOD* activity in leaves of olive trees as one of the most effective components of the antioxidant defense system against ROS toxicity (Mishra and Sharma 2019). However, the regulation of *SOD* is genotype-dependent, with stress tolerant cultivars showing a higher expression level (El Yamani and Cordovilla 2024). In LS, primed plants showed higher expression compared to control and non-primed plants, reinforcing the notion of a priming-induced protective effect. In contrast, CC showed the lowest *SOD2* levels in primed plants compared to the other treatments. These findings support genotype-specific responses to oxidative stress, with LS appearing to benefit from enhanced antioxidant defense against repeated stress, whereas CC appears to have a rapid but less prolonged response.

**Fig. 7:**
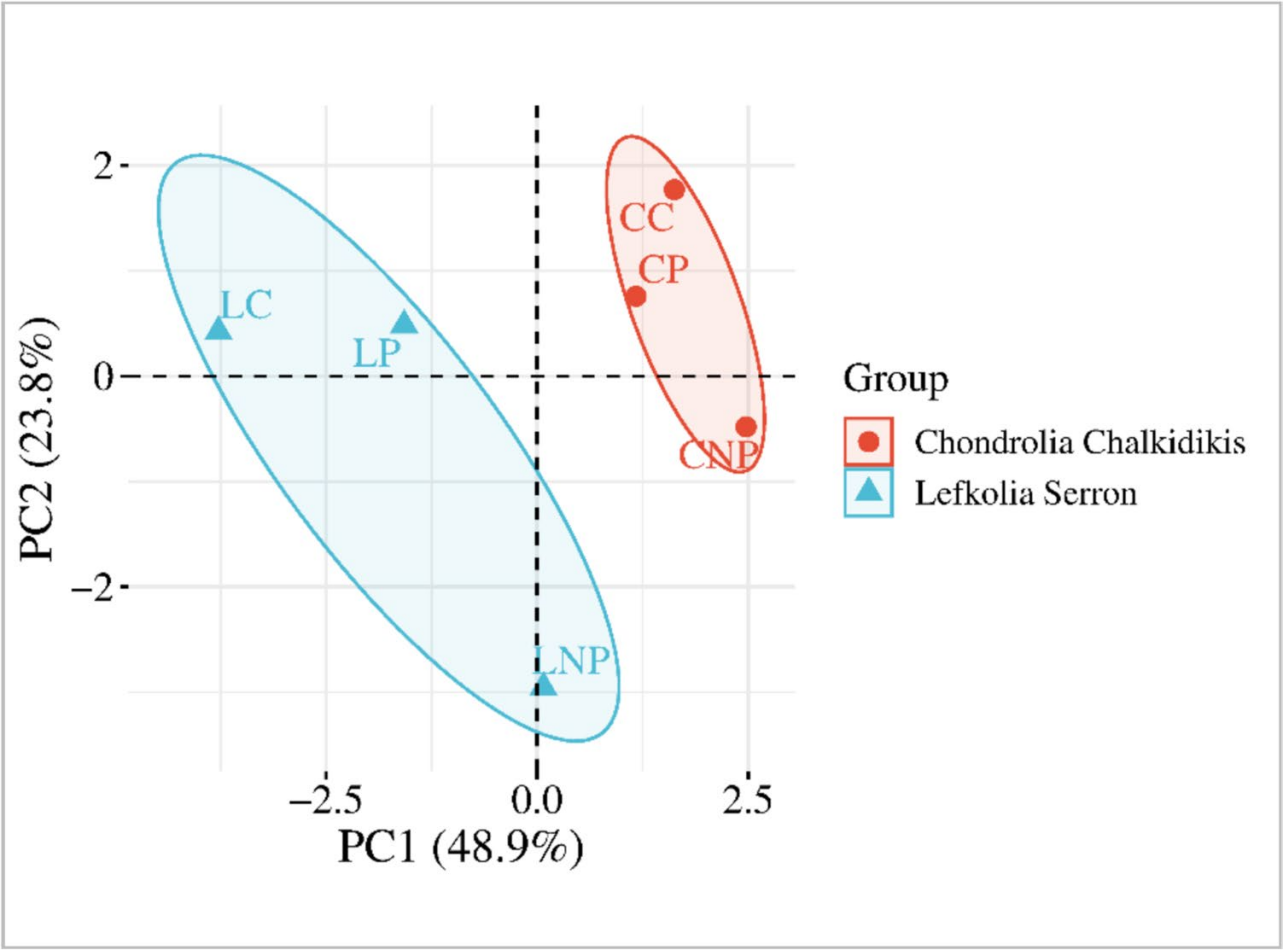
Principal Component analysis (PCA) of gene expression profiles at the final sampling point (T3). The plot illustrates the separation between the two olive cultivars of “Lefkolia Serron” and “Chondrolia Chalkidikis” among control (C), primed (P) and non-primed (NP) conditions, based on all analyzed genes. Samples of “Lefkolia Serron” are shown in blue (LC: Lefkolia control; LP: Lefkolia primed; LNP: Lefkolia non-primed), while samples of “Chondrolia Chalkidikis” are shown in red (CC: Chondrolia control; CP: Chondrolia primed; CNP: Chondrolia non-primed). The clustering pattern indicates genotype-specific differences in transcriptional responses, with “Lefkolia Serron” and “Chondrolia Chalkidikis” forming distinct groups. Control (C) and primed plants (P) of both cultivars appeared closer regarding non-primed plants (NP) where both appeared in the lower quadrant

Principal Component Analysis (PCA) was also performed to interpret gene expression data (Fig. 7). The results showed that PC1, which accounts for 48.9% of the total variance, separates the cultivars from each other, while based on PC2, 23.8% is due to the difference between treatments. An interesting result is that within each cultivar, the relative proximity of control and primed plants, suggests that priming induces a transcriptional condition that is more similar to the unstressed state. This is further emphasized by the grouping of non-primed plants away from the control and primed plants, suggesting a stronger shift in gene expression as a result of drought stress without prior exposure.

## Conclusion

In the present work, we investigated the response mechanisms of two Greek olive cultivars “Lefkolia Serron” and “Chondrolia Chalkidikis”, under water deficit and whether a prior stress could act as a priming agent. On priming studies, the experimental designs vary widely across plant species and stress types, with no standardized time frames, whereas the critical requirement is that the second stimulus is applied after recovery so that it is sufficient to elicit memory-related responses (Hilker et al. 2016; H. Liu et al. 2022a, b; Turgut-Kara et al. 2020; Vannay et al. 2026). In our study, morpho-physiological, metabolomic, and gene expression data reveal the drought strategies of the two cultivars.

In LS plants, long-term drought priming slightly reduced photosynthesis (T1) but enhanced later stress resistance (T3), shown by higher chlorophyll *α* fluorescence, iWUE, photosynthetic recovery, and upregulation of *MAPK5*, *ABARPYL4-9*, and *SOD2*. The metabolic profile of LS also indicated a consistent biochemical response to all treatments, implying a stress memory mechanism. CC plants showed a decrease in morpho-physiological performance during the initial drought period (T1) and a strong recovery upon re-watering (T2). In CC plants, priming benefits at T3 were limited: despite improved chlorophyll α fluorescence and iWUE, water loss persisted, indicating weaker drought acclimation than LS. Gene expression analysis showed a mild activation of drought responsive pathways, lacking associated physiological gain. Moreover, metabolomic profiling indicated a high metabolic activity in all treatments in CC which implies a re-active rather than a regulated or sustained acclimation response. The findings of our work highlight that long-term drought priming can be a promising approach to enhanced drought resilience in olive cultivars. However, its effectiveness appears to be genotype-dependent, underscoring the need for further research on molecular and physiological mechanisms. The different responses of CC and LS -two important Greek cultivars- to drought, highlight their distinct adaptive potential and confirm their importance for non-irrigated olive trees. The expression of drought priming and stress memory mechanisms further highlights their ability to cope with increasingly variable climates. This knowledge can be directly applied in nurseries and farmers to produce drought‑tolerant propagation material and guide selection in breeding programs. The use of such acclimatized genetic material could support farmers with more efficient and cost-effective management of crops, particularly under the challenges posed by climate change.

## Supplementary Information

Below is the link to the electronic supplementary material.

Supplementary file1 (DOCX 2203 kb)

Supplementary file2 (XLSX 32 kb)

Supplementary file3 (XLSX 12 kb)

Supplementary file4 (XLSX 12 kb)

Supplementary file5 (XLSX 12 kb)

Supplementary file6 (XLSX 11 kb)

## Data Availability

The datasets generated and/or analyzed during the current study are partly included in the supplementary information files. Additional datasets that support the findings of this study are available from the corresponding author on reasonable request.
