# Supplementary material for: Primed acclimation of two Greek olive cultivars to water deficit
Source: Physiol Mol Biol Plants. 2026 Apr 30;32(6):1309–24. doi: 10.1007/s12298-026-01747-x (PMC13291286; doi:10.1007/s12298-026-01747-x)
Supplement: Supplementary file 1 — Supplementary file1 (DOCX 2203 kb) [file 12298_2026_1747_MOESM1_ESM.docx]

*[Physiology and Molecular Biology of Plants](https://link.springer.com/journal/12298?IFA)*

**Primed acclimation of two Greek olive cultivars to water deficit**

**Supplementary Material**

**Online Resource 1**

**Metabolomic profiling**

The samples were ground in liquid nitrogen, and 300 mg of tissue was extracted with 1.4 mL of methanol. Adonitol (1 mg mL^−1^) was added as an internal quantitative standard, and samples were incubated for 10 min (70 °C). In the supernatant, 0.75 mL of chloroform and 1.5 mL dH_2_O were added and then centrifugated. An aliquot of 0.15 mL of supernatant was transferred into a vial glass and dried in a desiccator under vacuum. The residues were redissolved in 20 mg mL^−1^ methoxyamine hydrochloride and then in 0.07 mL N–methyl–N–(trimethylsilyl) trifluoroacetamide reagent (MSTFA) for 120 min and 30 min at 37 °C, respectively.

**Online Resource 2**

**Gene expression patterns**

Each reaction mixture included the following: 5 μL of PowerUp™ SYBR™ Green Master Mix, 0.4 μL for each of the forward and reverse primers, 2 μL of cDNA, and water to a final volume of 10 μL. The qPCR cycling program was set as follows: initial denaturation at 95°C for 3 minutes, amplification for 40 cycles at 95°C for 3 seconds, and extension for 30 seconds at 60°C. The specificity of the qRT-PCR reactions was evaluated by melting curve analysis, as follows: 95°C for 15 seconds and 1 minute at 60°C. Temperature was then gradually increased in 0.3°C intervals until it reached 95°C, with each step lasting 15 seconds.

**Table 1** Gene sequences used in qRT-PCR for gene expression analysis and their functional category. F and R indicate the forward and reverse primers, respectively.

| **Functional category** | **Primer Name** | **Sequences** |
| --- | --- | --- |
| Protein kinase | *MAPK5* | F: GAAGCCTGGGAACCTACTTATC |
|  |  | R: ACCATCGAGTGACCACATATTC |
| Transcription factor | *WRKY11* | F: CGCCGGAAACTGATGTTATTG |
|  |  | R: GGCCATCCAAACAAAGAGAATC |
| Membrane transport proteins | *AQP4* | F: CATCGGTGGGAGAGCTTATTG |
|  |  | R: ATGTACAGCGAGGAGGAGAA |
| Osmotic regulation | *DEH10* | F: CGTATGGAACTCATGGTGGAA |
|  |  | R: CGGGCAACCTCTCCTTTATC |
| ABA receptor | *ABARPYL4-9* | F: AGGCCTACAAGCACTTTCTC |
|  |  | R: CAACCACGCTAAAGCTCATAAC |
| Primary metabolism | *MI3PS2* | F: AAGGGCTAAGGTTCTGGATATTG |
|  |  | R: TTGTTCTTGCTTGGTTCCTTTG |
|  | *OesSUSY* | F: GCCTGGACTCTACCGAGTTGTT |
|  |  | R: CACGCATAGGTGTTCCTTGTTC |
| ROS scavenging | *SOD2* | F: GCCTCGACTGTTGTCAAGTTA |
|  |  | R: TTGTCGATAGCCCAACCTAAAG |
| Osmotic regulation | *ProDH* | F: GGCCTATTTGGGGAGTGGTAAA |
|  |  | R: CTACTGAGATGGCAATACAAGGAT |
| Stomatal development and cytokinesis | *STOMCYTDEF10* | F: CTCAGGAGAGAATTGGTGGTTTA |
|  |  | R: CATTACGCCCACCGCTATAA |
| Molecular chaperones | *STI1* | F: CAAGCGGATCTCTCCATTCTT |
|  |  | R: GGCTGCCCTTTCTGTATATCTT |

**Table 2*.*** Summary of qPCR standard curve parameters (slope, R² and amplification efficiency) for all genes included in the study.

| **Gene ID** | **Slope** | **R^2^** | **Efficiency (%)** |
| --- | --- | --- | --- |
| *MAPK5* | -3.125 | 0.941 | 108.91 |
| *AQP4* | -3.313 | 0.992 | 100.37 |
| *SOD2* | -3.218 | 0.958 | 104.52 |
| *STOMCYTD* | -3.383 | 0.864 | 97.50 |
| *STI1* | -3.133 | 0.977 | 108.54 |
| *WRKY11* | -3.181 | 0.997 | 106.23 |
| *DEH10* | -3.267 | 0.986 | 102.35 |
| *MI3PS2* | -3.357 | 0.923 | 98.57 |
| *ABARPYL4* | -2.947 | 0.999 | 118.42 |
| *OeSUSY* | -3.886 | 0.987 | 80.85 |
| *ProDH* | -3.529 | 0.982 | 92.02 |
| *EF1a* | -3.483  -3.377  -3.322 | 0.99  0.99  0.99 | 93.68  97.75  99.98 |

**Standard curves**


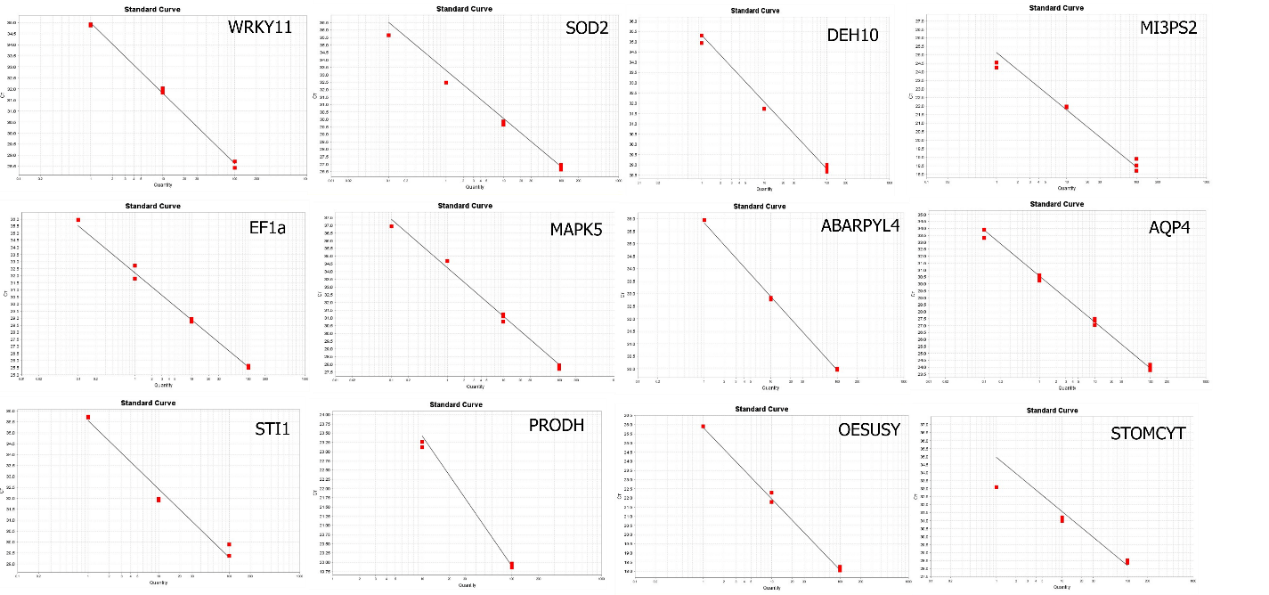


**Fig 1** Standard curves for all qPCR assays included in the study. Each curve was generated using a 4‑point, 10‑fold serial dilution

**Melt‑curve analysis**


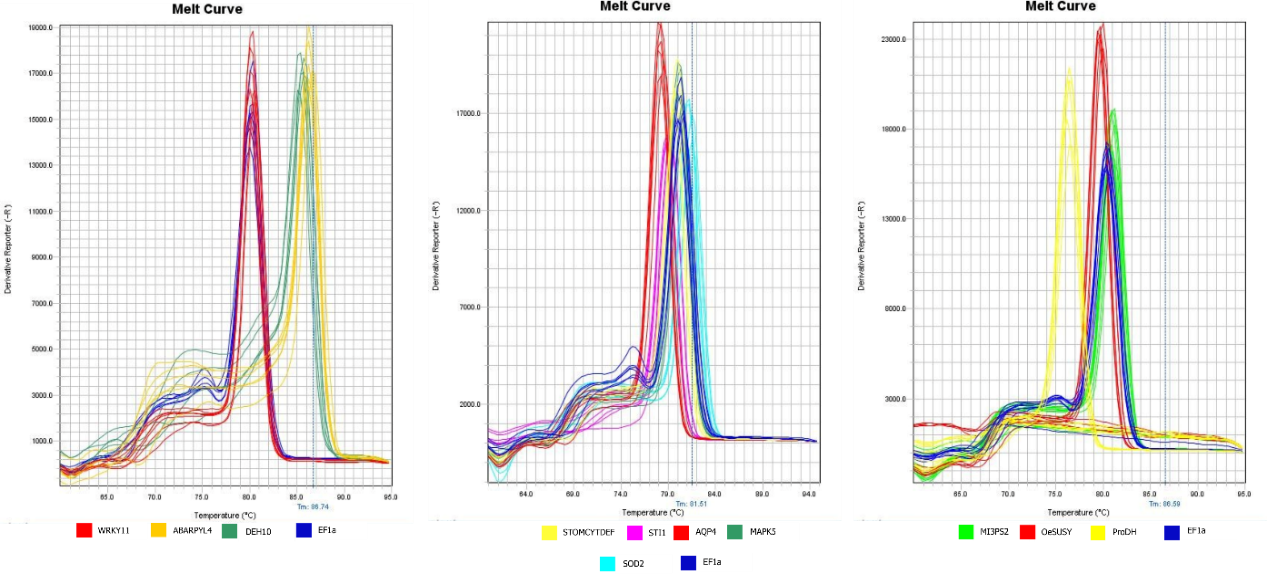


**Fig 2** Melt‑curve analysis for all qPCR assays included in the study

**Online Resource 3**

**Priming-dependent versus conserved metabolic responses in CC and LS olive plants**

**
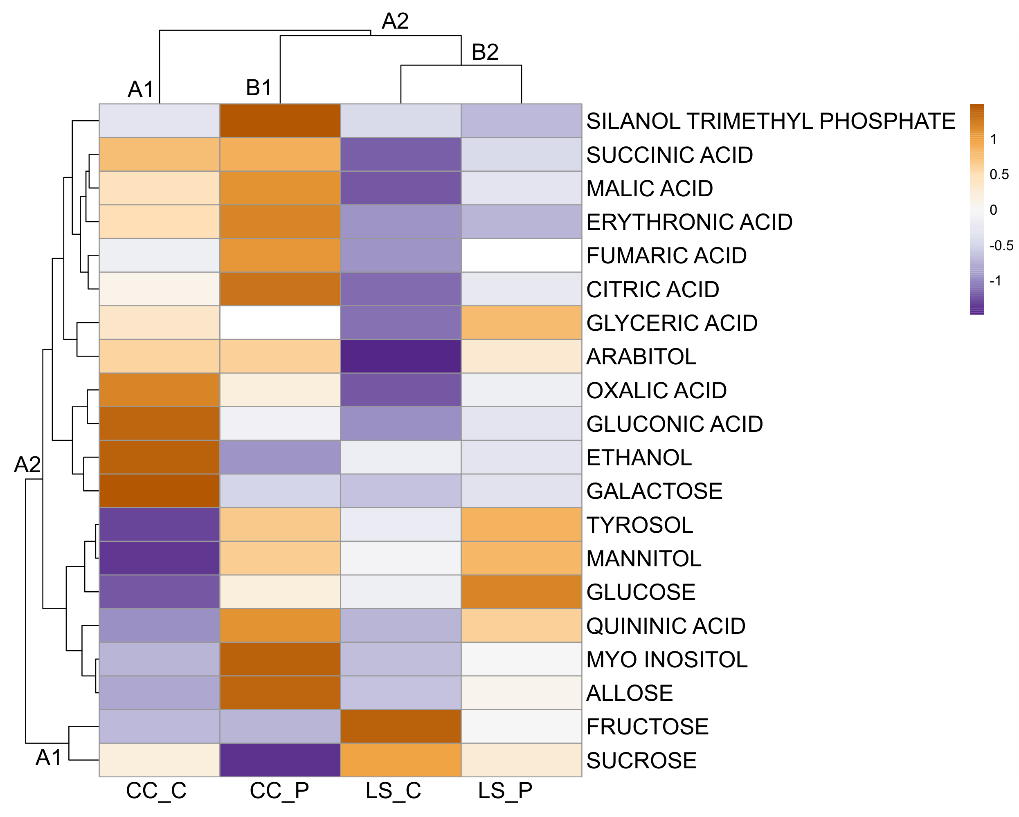
**

**Fig 1** Heatmap of primary metabolite changes in leaf samples collected at T1, (day 43), representing LS and CC among control (C) and primed (P) treatments. The heatmap illustrates the log₂-transformed relative abundance (1 mg/mL adonitol as internal standard) of primary metabolites, with blue indicating decreased metabolite levels and orange indicating increased metabolite levels across the different treatments. Hierarchical clustering is shown for both metabolites (left) and experimental groups (top). In total, 20 metabolites were identified, including five sugars, four sugars alcohols, nine organic acids, two alcohols and one other compound. The results reveal distinct metabolic differences between the LS and CC


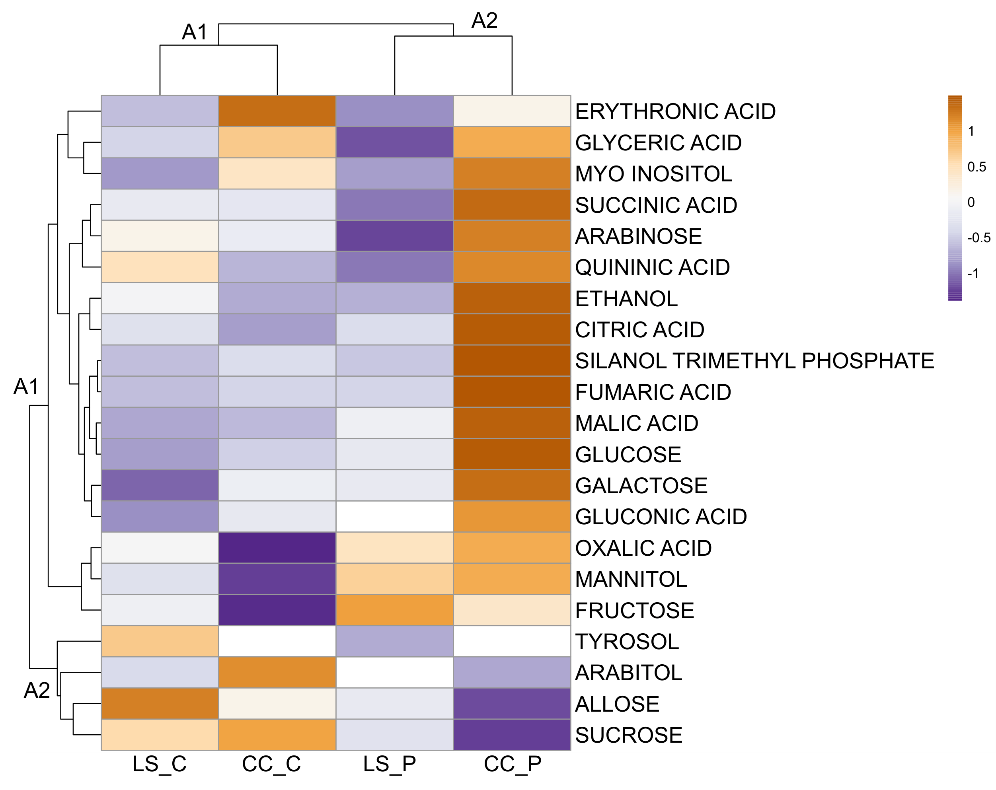


**Fig 2** Heatmap of primary metabolite changes in leaf samples collected at T2, (day 44), representing LS and CC among control (C) and primed (P) and treatments. The heatmap illustrates the log₂-transformed relative abundance (1 mg/mL adonitol as internal standard) of primary metabolites, with blue indicating decreased metabolite levels and orange indicating increased metabolite levels across the different treatments. Hierarchical clustering is shown for both metabolites (left) and experimental groups (top). Ιn total, 21 metabolites were identified, including six sugars, four sugars alcohols, nine organic acids, two alcohols and one other compound. Interestingly, 24 hours after watering, primed treatments grouped together

**Online Resource 4**

**Drought priming ‘restores’ gene expression in olive plants**

**
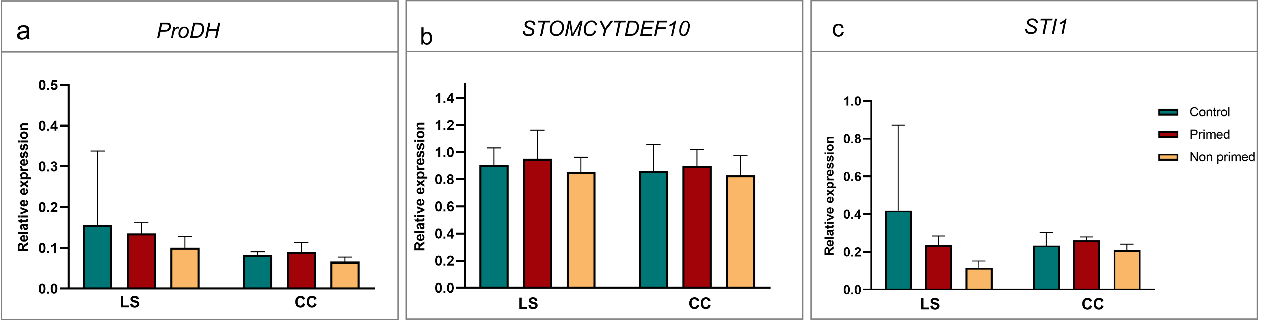
**

**Fig 3** Relative gene expression analyses of drought-related genes *ProDH*, *STOMCTDEF10* & *STI1* of cultivars LS and CC among control, primed and non-primed treatments at the final day of the experiment (T3). The bars represent the means and standard errors of three biological replicates. No statistically significant differences in the expression of these specific genes were observed between the cultivars or among the treatments
